# PacBio genome sequencing reveals new insights into the genomic organisation of the multi-copy *ToxB* gene of the wheat fungal pathogen *Pyrenophora tritici-repentis*

**DOI:** 10.1186/s12864-020-07029-4

**Published:** 2020-09-21

**Authors:** Paula Moolhuijzen, Pao Theen See, Caroline S. Moffat

**Affiliations:** Centre for Crop Disease and Management, School of Molecular and Life Sciences, Curtin University, Perth, WA Australia

**Keywords:** *Triticum aestivum*, Fungal pathogen, Tan spot, Necrotroph, Effector, Host-selective toxin

## Abstract

**Background:**

Necrotrophic effector proteins secreted by fungal pathogens are important virulence factors that mediate the development of disease in wheat. *Pyrenophora tritici-repentis* (Ptr), the causal agent of wheat tan spot, has a race structure dependent on the combination of effectors. In Ptr, ToxA and ToxB are known proteinaceous effectors responsible for necrosis and chlorosis respectively. While Ptr ToxA is encoded by the single gene *ToxA*, *ToxB* has multiple loci in the Ptr genome, which is postulated to be directly related to the level of ToxB production and leaf chlorosis. Although previous analysis has indicated that the majority of the *ToxB* loci lie on a single chromosome, the exact number and chromosomal locations for all the *ToxB* loci have not been fully identified.

**Results:**

In this study, we have sequenced the genome of a race 5 ToxB-producing isolate (DW5), using PacBio long read technology, and found that *ToxB* duplications are nested in the complex subtelomeric chromosomal regions. A total of ten identical *ToxB* gene copies were identified and based on flanking sequence identity, nine loci appeared associated with chromosome 11 and a single copy with chromosome 5. Chromosome 11 multiple *ToxB* gene loci were separated by large sequence regions between 31 and 66 kb within larger segmental duplications in an alternating pattern related to loci strand, and flanked by transposable elements.

**Conclusion:**

This work provides for the first time the full accompaniment of *ToxB* loci and surrounding regions, and identifies the organization and distribution of ten *ToxB* loci to subtelomeric regions. To our knowledge, this is the first report of an interwoven strand-related duplication pattern event. This study further highlights the importance of resolving the highly complex distal chromosomal regions, that remain difficult to assemble, and can harbour important effectors and virulence factors.

**Supplementary information:**

**Supplementary information** accompanies this paper at 10.1186/s12864-020-07029-4.

## Background

The inverse gene-for-gene interactions between host plants and necrotrophic fungal pathogen typically involve pathogen effectors, which interact with a compatible locus in the host leading to toxin sensitivity and disease susceptibility.

*Pyrenophora tritici-repentis* (Ptr) a necrotrophic fungal pathogen and the causal agent of wheat (*Triticum aestivum* L) tan spot, produces a number of effectors that mediate the development of foliar disease on susceptible wheat genotypes. Tan spot has two distinct leaf symptoms, which are necrosis and chlorosis [1]. These symptoms are the result of secreted effectors ToxA, ToxB and ToxC [2–4] and other as yet uncharacterised effectors [5, 6]. ToxA and ToxB, are characterised as small effector proteins that produce necrosis and chlorosis symptoms, respectively [2, 4]. While ToxC, which also causes chlorosis, has not been characterised and may be the product of a secondary metabolite gene cluster [3].

For the two proteinaceous toxins, ToxA reacts with a corresponding susceptibility gene in wheat (*Tsn1*), which makes the host sensitive to the effector [7], while the corresponding host gene for ToxB remains as yet unknown but is associated with the *Tsc2* locus on chromosome 2B [8].

In the Ptr genome, *ToxA* is a single locus gene, the result of a horizontal gene transfer from another fungal pathogen species [9]. While in contrast, there are multiple identical gene copies of *ToxB* [10, 11], in which the copy number variation has been shown to have an association with virulence. Nine copies of *ToxB* in race 5 isolates (DW2, DW7, DW13 and DW16), were estimated by phosphoimage analysis, and of these six copies were individually cloned and sequenced from DW7 (1-3 kb in length) [10]. Southern analysis indicated that the *ToxB* loci reside on two unknown chromosomes, approximately 3.5 and 2.7 Mb in length, with the majority located on the smaller chromosome [10].

To date a number of Ptr whole genome sequencing projects involving race 5 isolates (ToxB-producing) have not been able to determine if the *ToxB* loci are clustered or dispersed [12, 13] in the genome. We therefore undertook genome sequencing via PacBio long read technology to resolve the number, organization and distribution of *ToxB* loci within the genome of a race 5 isolate (DW5). A comparative analysis of these *ToxB* regions to a race 1 isolate (ToxB non-producing), which was previously assembled from PacBio long read technology and optical mapping [12], was undertaken to identify any flanking sequence conservation.

## Results

### Ptr isolate DW5 whole genome assembly analysis

The Ptr race 5 isolate DW5 was sequenced using long read single molecule PacBio technology and the error corrected reads were assembled and annotated (Table 1). Furthermore, a previous PacBio sequenced Ptr race 1 isolate (M4), which was scaffolded into chromosomes based on an optical map, but not annotated at the time [12], was also annotated during this study. The DW5 genome assembly size was 40.87 Mb, close to the genome size of M4 at 40.92 Mb [12], however DW5 was slightly more fragmented with 60 contigs as compared to the 50 contigs for M4 [12]. This fragmentation may be directly related to a slightly higher repeat content in DW5 and the slightly smaller content of protein coding genes compared to M4 (Table 1). Protein coding gene predictions for the DW5 contigs and M4 scaffold assemblies were 14,276 and 15,466, respectively. The DW5 annotated genome has been deposited at DDBJ/ENA/GenBank under the accession MUXC00000000. The version described in this paper is version MUXC02000000. The annotated M4 genome has been deposited in DDBJ/ENA/GenBank under accession NQIK00000000. The version described in this paper is version NQIK02000000.

**Table 1:** Ptr isolate sequence, assembly and annotation statistics

	M4^a^	DW5
*Isolate information*		
Race	1	5
Effectors	AC	B
Collection site	Western Australia	North Dakota
Collection year	2009	1998
*Sequencing*		
Sequencing Platform	PacBio RSII	PacBio Sequel
Number of reads	594,877	1,306,274
Total sequence (Mbp)	3500	14,225
Sequencing coverage	75	355.62
*Error correction*		
Number of reads	56,338	302,147
Error corrected sequence (Mbp)	758	3085
Genome coverage	19	77.13
*Assembly statistics*		
NCBI accession	NQIK00000000	MUXC00000000
Number of contigs	50	60
Number of scaffolds*	41*	NA
Total length contigs/scaffolds* (Mb)	40.92*	40.87
N50 (Mb)	3.65	3.13
L50	4	5
Mean size (kb)	998	681
GC %	50.73	50.21
Repeat %	7.96	8.33
*Gene predictions*	M4^b^	
Gene number	15,443	14,276
Total CDS (Mb)	20.7	17.9
Average size (kb)	1.3	1.2
*Predicted effectors*		
Number of predicted effectors	445	401
Total length (aa)	61,737	60,150
Mean gene size (aa)	138	150
Median gene size (aa)	117	123

^a^ Genome was sourced from previous optically mapped assembly [12]. ^b^ M4 genome annotation this study. * scaffolds

### Whole genome comparative analysis between Ptr races 1 and 5

**Fig. 1:**
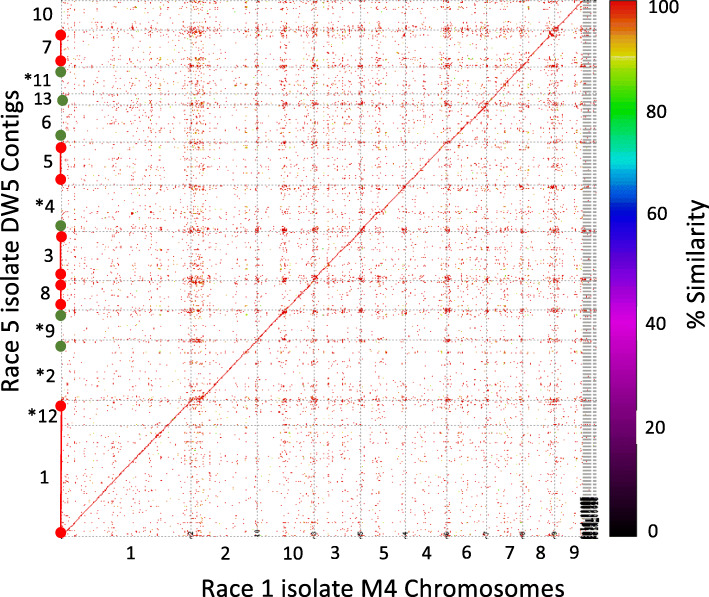
Ptr nucleotide sequence alignment for race 5 isolate DW5 contigs (vertical axis) to race 1 M4 chromosomes (horizontal axis). The sequence dot plot shows the percent sequence similarity between the two genomes. The diagonal red line is the maximal colinear alignment between the two genomes. Individual contigs and chromosomes are delineated by grey lines in both axes of the sequence dot plot. *DW5 contigs are reverse complemented. On the vertical axis, DW5 contigs that represent chromosomes are informed by telomere repeat motifs, single (green circle) and both (red circles connected by a red bar)

The genome sequence of DW5 (race 5) was aligned to M4 (race 1) [12] to determine sequence conservation at a chromosome level. Thirteen DW5 contigs showed colinear alignment to the scaffolded M4 chromosomes at greater than 98% sequence identity (Fig. 1) with no large-scale chromosomal rearrangements. DW5 contigs 3, 5, 7 and 8 were sequenced from 5′ telomere to 3′ telomere informed by the presence of the tandem telomere repeat motifs (CCCTAA)n/(TTAGGG)n.

Based on M4 chromosomes, thirteen DW5 assembled contigs matched nine chromosomes, which included chromosomes 1–9 (Table 2). A chromosome fusion between chromosome 10 and 11 (referred to as chromosome 10) in Australian isolate M4 resolved by optical mapping [12] was not observed for DW5, where DW5 contig 8 possessed both 5′ and 3′ telomere motifs (Table 2), which would represent chromosome a (telomere to telomere).

**Table 2:** DW5 genome assembly relative to M4

M4 chromosome	M4 chromosome length (Mb)	DW5 contig	DW5 contig length (Mb)	DW5 contig 5′ and 3′ telomere motifs
1	9.91	1 12^a^	8.11 1.82	Yes
2	5.07	2^a^	4.42	No
3	3.65	3	3.65	Yes
4	3.15	5	3.13	No
5	3.38	4^a^	3.36	Yes
6	3.05	6	2.73	No
7	2.76	13 11^a^	0.83 1.96	Yes
8	2.40	7	2.68	Yes
9	2.17	10	2.12	No
10	4.30	9^a^	2.18	No
10	4.30	8	2.18	Yes

^a^Reverse complemented sequence

### Multiple *ToxB* loci have alternate strand positions

**Fig. 2:**
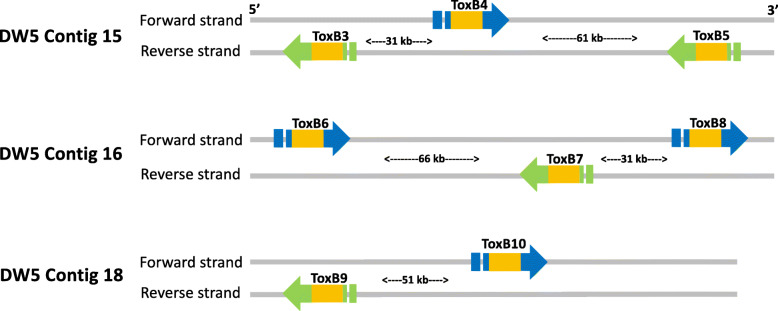
Alternating strand positions of *ToxB* loci Ptr isolate DW5 in contigs 15, 16 and 18. *ToxB* loci are shown as blue arrow on the forward strand and green on the reverse strand. The coding sequence is shown in yellow

The DW5 assembly was searched for *ToxB* homologs and 10 copies were identified across 5 contigs (DW5_contig_0004, DW5_contig_0009, DW5_contig_00015, DW5_contig_00016 and DW5_contig_00018). A single *ToxB* loci was found for each of the larger two contigs DW5_contig_0004 (3.65 Mb) and DW5_contig_0009 (2.18 Mb), labelled here as ToxB1 and ToxB2, respectively (Table 3). Multiple *ToxB* loci were located on the smaller contigs DW5_contig_00015 (ToxB3, ToxB4 and ToxB5), DW5_contig_00016 (ToxB6, ToxB7 and ToxB8) and DW5_contig_00018 (ToxB9 and ToxB10), sized 126, 123 and 99 kb, respectively. *ToxB* genes were not immediate neighbours and loci appeared to locate in alternate strand positions separated by relatively large distances that ranged between 31 and 66 kb in size. This pattern was observed across the three contigs (DW5_contig_00015, DW5_contig_00016 and DW5_contig_00018) harbouring multi-loci *ToxB* (Fig. 2).

**Table 3:** Ptr isolate DW5 *ToxB* subtelomeric gene locations, chromosomes relative to M4 and sequence identity to DW7 *ToxB* cloned sequence

Ptr DW5							Ptr M4	Ptr DW7		
***ToxB***^a^	Contig	Contig size (Mb)	***ToxB*** Gene start	***ToxB*** Gene end	***ToxB*** Strand	Loci Label	M4^**b**^	DW7^**c**^	DW7 cloned sequence length (bp)	DW5 ***ToxB*** locus % Sequence identity^d^
A1F99_069381	DW5_contig_00004	3.365	3,306,588	3,306,848	+	ToxB1	Chr5	AY425485 (ToxB6)	1769	100.00
A1F99_114980	DW5_contig_00009	2.180	2,152,566	2,152,826	–	ToxB2	Chr10	AY425481 (ToxB2)	3563	99.99
A1F99_139310	DW5_contig_00015	0.126	10,557	10,817	–	ToxB3	None	AY425481 (ToxB2)	3563	99.99
A1F99_139400	DW5_contig_00015	“”	41,790	42,050	+	ToxB4		AY425480 (ToxB1)	4471	100.00
A1F99_139580	DW5_contig_00015	“”	103,025	103,285	–	ToxB5		AY425483 (ToxB4)	1696	100.00
A1F99_139650	DW5_contig_00016	0.123	4627	4887	+	ToxB6	None	AY425484 (ToxB6)	2494	100.00
A1F99_139840	DW5_contig_00016	“”	70,550	70,810	–	ToxB7		AY425480 (ToxB1)	4471	99.44
A1F99_139950	DW5_contig_00016	“”	112,439	112,699	+	ToxB8		AY425480 (ToxB1)	4471	100.00
A1F99_140280	DW5_contig_00018	0.099	11,326	11,586	–	ToxB9	None	AY425483 (ToxB4)	1696	100.00
A1F99_140440	DW5_contig_00018	“”	62,388	62,648	+	ToxB10		AY425482 (ToxB4)	4007	100.00

^a^ DW5 GenBank locus tag number, ^b^ Contig alignment to M4 chromosomes, ^c^ DW7 cloned *ToxB* genome sequence GenBank accessions [10], ^d^ DW5 percent sequence identity to DW7 *ToxB* sequences [10]

### Multiple *ToxB* loci are associated with subtelomeric chromosomal regions

**Fig. 3:**
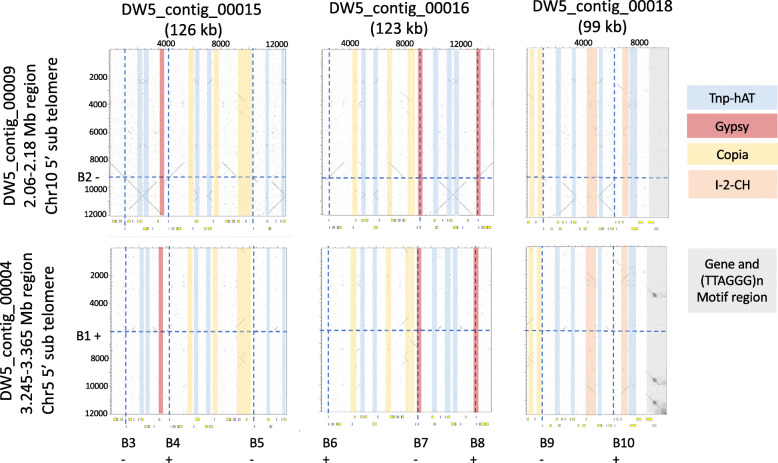
DW5 *ToxB* contig nucleotide sequence alignments to terminal chromosome regions. DW5 sub-telomeric regions (120 kb) for contig 4: 3,245,792-3,365,792 bp (chromosomes 5) and contig 9: 2,060,091-2,180,091 bp (chromosome 10) (vertical axis). DW5 contigs 15, 16 and 18 are represented on the horizontal axis with gene annotations below (yellow boxes). Sequence alignments are plotted in the sequence plot in grey. *ToxB* loci positions for chromosome 5 and 10 are indicated with horizontal blue dashed lines for B1 and B2. The remaining locations of *ToxB* in the contigs are shown with vertical dashed blue lines for contigs 15, 16 and 18. To view any repeat element patterns major elements are highlighted for contigs 15, 16 and 18, as Tnp-hAT transposons, Gypsy and Copia retrotransposons, *Bipolaris maydis* I-2-CH element and a region containing the (TTAGGG)n telomere motif

**Fig. 4:**
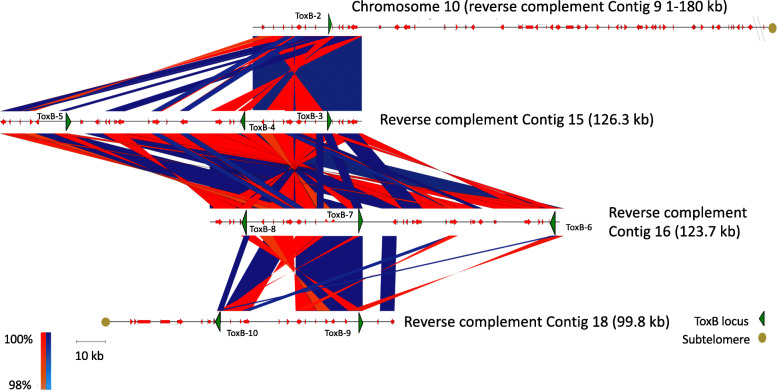
DW5 contig sequence homology between 5′ subtelomere regions of chromosome 10 and *ToxB* loci. Nucleotide sequences are from top to bottom, chromosome 10 (zoomed on reverse complemented contig 9:2.0–2.18 Mb), contigs 15, 16 and 18. *ToxB* loci (green triangles) and surrounding genes (red arrows) are shown for each contig. Telomere motifs (brown circles) are shown on the 3′ and 5′ end of chromosome 10 and reverse complemented contig 18, respectively. Sequence homology between contigs is shown on the same strand (blue) and complementary (red)

Based on genome alignments to M4, two contigs (DW5_contig_0004 and DW5_contig_0009) with single *ToxB* loci were syntenic with the subtelomeric regions of M4 chromosomes 5 and 10, respectively (Fig. 1 and Table 3). No significant alignments were identified for the three smaller multiple *ToxB* loci contigs (DW5_contig_0015, DW5_contig_0016 and DW5_contig_0018) to the genome of M4. However, a search back to the DW5 genome (self-search) identified alignments for all three contigs to chromosome 10 (DW5_contig_0009) (Fig. 3), sequence breaks can be seen where regions of paralogous sequence are interspersed with repeat elements. No other alignments to the DW5 genome were found except for self-contig alignments. The alignment of the fragmented *ToxB* contigs with the 5′ subtelomeric region of chromosome 10 (reverse complemented DW5_contig_0009) and the presence of a 5′ telomere motif (TTAGGG)n in chromosome 5 (reverse complemented DW5_contig_0004) (Table 4), weighted chromosome 10 as the possible origin of ToxB3–10 loci and chromosome 5 (DW5_contig_0004) as the only source for the ToxB1 locus*.* The alignment of the 5′ telomere region of chromosome 10 and *ToxB* loci (ToxB3 to ToxB10) thus implied that contigs 15, 16 and 18 could be the fragmented regions not assembled from the 5′ telomere region of chromosome 10 (Fig. 4).

**Table 4:** *ToxB* contigs and telomere motifs

Contig	Contig size (Mb)	5′ Motif (ccctaa)n	3′ Motif (ttaggg)n	M4^a^
DW5_contig_00004 (reverse complement)	3.365	+	–	Chr5
DW5_contig_00009 (reverse complement)	2.180	–	+	Chr10
DW5_contig_00015	0.126	–	–	None
DW5_contig_00016	0.123	–	–	None
DW5_contig_00018	0.099	–	+	None

^a^ M4 chromosome with DW5 *ToxB* contig alignment

All *ToxB* loci, except ToxB6, which was truncated in the 5′ region upstream of *ToxB*, were co-located with dimer Tnp-hAT repeat genes. The dimer Tnp-hAT genes were located 10–15 kb upstream of the *ToxB* loci.

### Larger groups of conserved regions are found between the *ToxB* loci based on strand positions

**Fig. 5:**
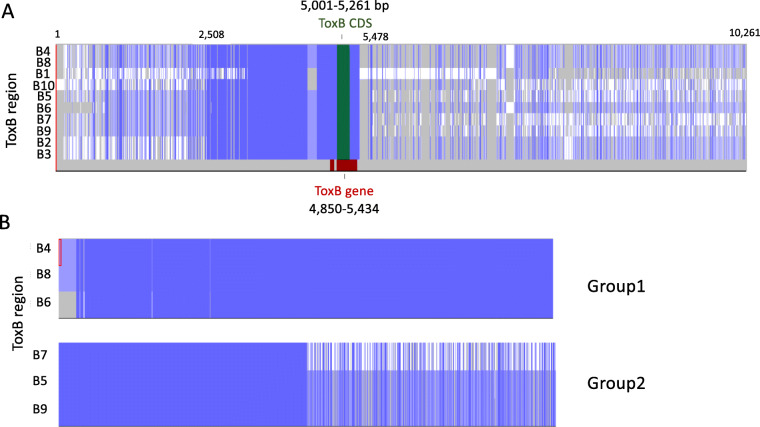
Multiple nucleotide sequence alignment of the ten *ToxB* 10 kb regions. **a** Conserved sequence (blue), sequence not conserved (white) and sequence gaps (grey) are shown for the ten *ToxB* 10 kb regions. A highly conserved region (2508–5478 bp) was mainly upstream of *ToxB* (red) and the complete coding sequence (CDS) (green). **b** Sequence alignment overview (not to scale) show the 10 kb *ToxB* region conserved sequences grouped by the locus strand. Group 1 (B4, B6 and B8) forward strand *ToxB* loci and Group 2 (B5, B7 and B9) reverse strand *ToxB* loci

The *ToxB* loci and flanking sequence regions of 5 kb upstream and downstream were extracted (including *ToxB* mRNA transcript) for a nucleotide multiple sequence alignment to determine sequence conservation between the ten loci. Only ToxB6 was truncated in the 5′ sequence region due to the locus location (contig16:4,627–4,887 bp). The *ToxB* 10 kb multiple sequence alignment showed a highly conserved region of 3,170 bp with a large proportion (2.5 kb) highly conserved upstream of *ToxB* for all ten loci (Fig. 5a). On closer examination, the *ToxB* 10 kb regions could be grouped by their locus strand (Fig. 5b). The full 10 kb regions were highly conserved for *ToxB* loci B4, B6 and B8 on the forward strands of contigs 15 and 16 (group 1). While further conservation was found for reversed stranded *ToxB* loci B5, B7 and B9 (group 2) on contigs 15, 16, and 18, and to a lesser extent for reverse strand *ToxB* loci B2 and B3 (group 3) on contigs 9 and 15 (not shown in Fig. 5).

**Fig. 6:**
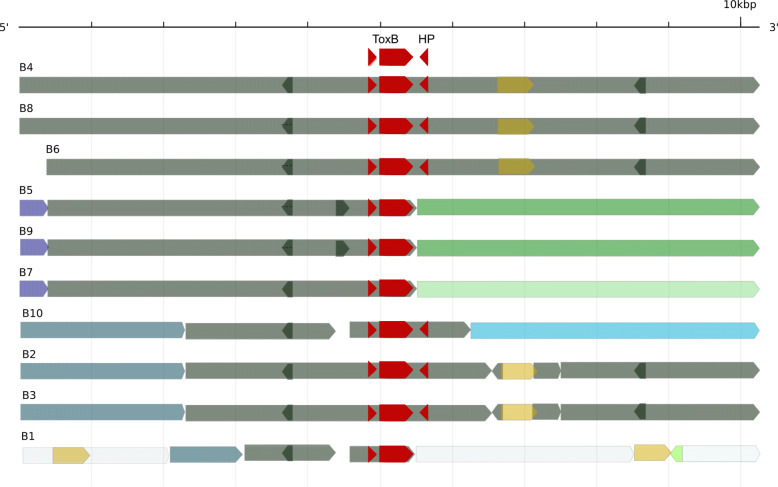
DW5 *ToxB* 10 kb locus region (B1-B10) sequence homology. Regions in common are represented by the same colours relative to the *ToxB* gene position (red). For example, a small 5′ region is shared by B5, B7 and B9 and is shown in purple. Grey regions are common to all ten loci and gaps are shown in white. The hypothetical protein (HP) is found downstream of *ToxB* in B2–4, 6, 8 and 10. Regions of similarity are shown between forward sequence (forward arrow) and reverse sequence (reverse arrow). Short regions of similarity contained within a larger region are secondary alignments

When the homology between the ten *ToxB* 10 kb regions was summarized for conserved and distinctive regions (Fig. 6), the 10 kb regions surrounding *ToxB1* on chromosome 5 were found to be more divergent than the remaining loci proposed to be from chromosome 10. It was also noted that a small hypothetical protein (128 bp) was conserved 288 bp downstream of the *ToxB* loci in all forward stranded positions except *ToxB1* and only in reverse positioned *ToxB2* and *ToxB3*.

### *ToxB* and promoter region

All ten copies of the 261 bp ToxB protein coding sequence are identical, as found previously for six of the sequenced copies [10]. Based on DW5 mRNA transcript from a previous study [12], *ToxB* has a two exon gene structure of 533 bp in length. *ToxB* exon1 (94 bp) and exon 2 (439 bp) flank an intron 52 bp in size. The exon 1 5′ UTR and exon 2 3′ UTR have lengths of 99 bp and 172 bp, respectively (Additional file 1). Previously, the *ToxB* promoter was reported to be greater than 300 bp upstream of the coding sequence [10]. The upstream region from *ToxB* (2 kb) was then searched for transcription binding site motifs. A DNA binding site was predicted upstream of *ToxB*, 847 bp from the starting codon of *ToxB2–9*, and 644 bp for *ToxB1* and *ToxB10* at an expected value of 4.9e-178. The most significant motif profile MA0320.1 (IME1) was identified with a probability value of 2.20e-06 (Additional file 2).

## Discussion

### Ptr ToxB multiloci analysis

**Fig. 7:**
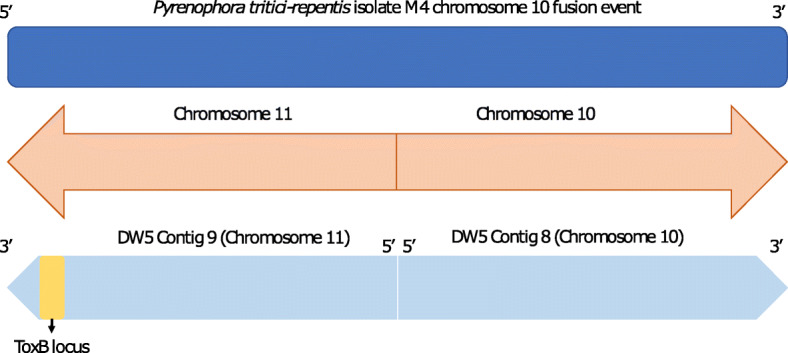
Overview of *ToxB* locus in DW5 relative to the M4 chromosome 10 fusion event. M4 chromosome 10 (top) is the result of a fusion between chromosomes 10 and 11 (shown in the middle). DW5 Contig 9 (chromosome 11) and Contig 8 (chromosome 10) (bottom) are shown relative to M4 chromosome 10. ToxB locus (yellow) which aligns to the 5’ distal region of M4 chromosome 10 is shown in the 3’ distal region of DW5 Contig 9 (chromosome 11)

This is the first genome sequence investigation into the distribution of *ToxB* loci in Ptr using long read sequencing technologies. A previous study for race 5 isolate DW7 found that six of the sequenced copies, all had identical protein coding sequence identity [10]. In this study, all the the *ToxB* loci (585 bp) identified have identical sequence, including exon and intron sequences. It was previously suggested that DW7 *ToxB* loci resided on two unknown chromosomes, approximately 3.35 and 2.7 Mb in size, with the majority of the loci on the smaller chromosome [10]. In this study, the *ToxB* loci were located on chromosome 5 and 11, which had assembly sizes of 3.36 and 2.18 Mb respectively, which are close to the previously estimated chromosome sizes by Martinez et al., (2004). Of the ten *ToxB* loci, nine appeared to be associated with the smaller chromosome 11 located in the 3′ distal region. A Ptr chromosome noted for a chromosome fusion event for a race 1 isolate M4 [12]. The telomere to telomere support for eleven DW5 chromosomes is similar to the findings for another American race 1 isolate Ptr Pt-1C-BFP [13], unlike the 10 chromosome genome of Australian isolate M4 [12] (Fig. 7). Large scale segmental rearrangements have been frequently identified in the subtelomere regions of fungal chromosomes, where breakage/fusion events and large-scale rearrangements frequently occur [12, 14, 15]. During meiosis the subtelomeric regions have instability often referred to as plasticity [16]. In these regions, chromosome breakage fusion cycles begin with the loss of telomeres which causes instability and potential fusion of sister chromatids. During the breakage fusion cycle, the site of breakage during separation in erroneously fused sister chromatids can lead to sequence duplication, deletion and rearrangement [16]. It is therefore probable that the recent highly conserved duplications of loci in race 5 have occurred through multiple breakage fusion events between the distal chromosome regions and may have at one stage been potentially lost from race 1 isolates.

Genome plasticity in distal chromosome regions can contribute to rapid fungal diversification, especially for Ptr [11]. In this study the subtelomeric *ToxB* loci location within Ptr DW5 provided a favourable environment for duplication, which may have provided this isolate a potential advantage for survival.

### Ptr ToxB patterns of duplication

In addition to the positioning of the *ToxB* duplication within the distal region of chromosome 11, *ToxB* loci were located equidistant downstream from dimer Tnp-haT transposases, a familiar gene found coupled to Ptr *ToxA* and within the horizontally transferred region, also found in *Parastagonospora nodorum* and *Bipolaris sorokiniana* [9, 17]. It is therefore possible that the dimer Tnp-hAT transposases observed in DW5 may have played a self-complementing role in the duplication of *ToxB*, providing regions of homology between flanking regions, resulting in larger regions of homology as observed between the multiple DW5 *ToxB* copies. Our data found that multiple *ToxB* gene duplication events involved much larger segmental duplications, flanked by transposable elements, than previously identified [10]. Here, we also identified that larger homologous regions could be grouped by the strand from which the duplicated *ToxB* is transcribed. Furthermore, we believe this is first reporting of a potential interwoven strand-related duplication pattern/event of a necrotrophic effector gene.

### *ToxB* transcription factor binding site analysis

The binding of transcription factors to specific DNA binding sites (identified by a DNA motif) is key for the transcriptional regulation of genes, here a transcription factor binding motif IME1 profile was identified upstream of the multiple *ToxB* loci. The motif of IME1 is a conserved regulatory site for *Saccharomyces cerevisiae*, previously identified from ChIP-chip data [18]. Although the IME1 transcription factor protein (UniProt accession P21190) is required for sporulation and early sporulation-specific genes expression, further experimental validation would be required in Ptr race 5 isolates to determine if the potential transcription factor is indeed involved in the regulation of *ToxB*.

## Conclusions

Our findings provided insights into the unique nature of the multicopy *ToxB* organisation in the Ptr genome and revealed a potentially complex effector gene regulatory network. This study directly works towards a better understanding of genome plasticity events in fungal adaptation and effector gene evolution.

## Material and methods

### Ptr race 5 isolate DW5 collection and sequencing

The Ptr race 5 isolate DW5 was collected in 1998 from North Dakota, USA and was kindly provided by Tim Friesen (North Dakota, USA).

Isolate genomic DNA was extracted from 3-day old mycelia grown in Fries 3 medium using the BioSprint 15 automated workstation according to the manufacturer’s instruction (Qiagen, Germany). DNA was then treated with 50 μg/ml of RNase enzyme (Qiagen, Hilden, Germany) for 1 h followed by phenol/chloroform extraction. DNA was precipitated with sodium acetate and ethanol, and resuspended in TE buffer [14].

The DW5 genome was sequenced using PacBio Sequel technology (https://www.pacb.com) by Novogene (China, https://en.novogene.com/). The PacBio sequence coverage for isolate DW5 was 77x. The DW5 genome was also Illumina sequenced (www.illumina.com) for 150 PE reads at 100x coverage by Novogene (China, (https://en.novogene.com/)). The Illumina data was used for post-genome assembly error correction (polishing).

### Ptr isolate DW5 whole genome assembly

The DW5 PacBio sequence data was error corrected and assembled using Canu version 1.9 [19] with pacbio-raw and genome size of 40 Mb parameter settings on a heterogeneous Hewlett Packard Enterprise Linux cluster (Zeus, https://pawsey.org.au). The DW5 assembled PacBio contigs were then indexed using BWA index version 0.7.17-r1188 [20]. The DW5 genomic Illumina read data, sequenced in this study, was then aligned to the indexed DW5 assembled PacBio contigs using BWA mem version 0.7.17-r1188 [20] (−t 16). The alignment file (BAM format) was then filtered for concordant read alignments using SAMTools version 1.7 view (−f 0 × 2) and sorted [21] for further genome error correction (polishing). The DW5 PacBio assembly was then error corrected using Pilon version 1.23 [22] (--changes --tracks --output DW5_pilon --defaultqual 20 --threads 16 --frags ‘DW5 sorted BAM file’).

The DW5 PacBio assembled genome was then masked for low complexity sequence and known fungal repeats using RepeatMasker (RM) [23] version 2.9.0+, Dfam 3.0 [24, 25] and Repbase 20,181,026 [26] with taxon fungi parameter available through a docker image (https://hub.docker.com/r/taavipall/repeatmasker-image).

### DW5 and M4 gene prediction and annotation

The PacBio DW5 assembled contigs and a previously assembled Ptr race 1 isolate M4 scaffold assembly [12] were indexed using bowtie2-build version 2.3.4.1 [27]. Previously sequenced stranded RNA-seq Illumina read data [12] for DW5 and M4 were aligned to the respective indexed genomes DW5 (DDBJ/ENA/GenBank accession MUXC02000000) and M4 (DDBJ/ENA/GenBank accession NQIK02000000) using TopHat2 version 2.1.1 [28] (--no-discordant -N 0 -i 10 -I 5000 -p 16 --library-type fr-firststrand). Based on the accepted TopHat2 alignments (BAM file), mRNA transcripts, in GTF format, were then generated using CuffLinks version 2.2.1 [29] (-p -library-type fr-firststrand). The transcript GTF file format was then converted to GFF3 using GenomeTools gtf_to_gff3 version 1.5.10 [30] to provide transcript support (evidence) towards the *ab initio* gene predictions.

*Ab initio* gene predictions were made with GeneMark-ES v 4.33 (--ES --fungus --cores 16 --evidence) [31] and Coding Quarry v2.0 [32] (-p 16 -t) in pathogen mode (PM), both *ab initio* gene predictions were supported by the transcript GFF3 file. Published Ptr protein FASTA sequences were downloaded from NCBI using NCBI txid45151 on the 20th January 2020 and aligned to the genomes using Exonerate v2.2.0 [33] (--showvulgar no --showalignment no --minintron 10 --maxintron 2000 --percent 90) mode protein2genome. The final gene prediction sets were then merged via EvidenceModeller v1.1.1 [34] using a combination of protein alignments and the two *ab initio* predictions on the genome, with a minimum intron length of 10 bp and evidence weights [31] CodingQuarry:10, GeneMark.hmm:10, Exonerate:5 and CuffLinks:10.

Gene annotations were assigned from BLASTX (v2.2.26) [35] searches (expected value ≤ 1e-05) against the following databases Uniref90 (October, 2019), NCBI Refseq (taxon = Ascomycota) (October, 2019) and sequence domains were assigned by RPS-BLAST (v2.2.26) against Pfam (October, 2019), Smart (October, 2019) and CDD (October, 2019). The blast protein and domain searches were then summarised using AutoFACT version 3.4 [36].

The annotated proteins were searched for signal peptides using SignalP version 5.0b [37] (-format short -gff3 -mature -org euk). Those identified with signal peptides were then searched for predicted effectors using EffectorP version 2.0 [38]. EffectorP 2.0 has a low false positive rate of 11.2% and a high accuracy of 88.8% for effector prediction [38].

### DW5 ToxB identification and analyses

All published ToxB sequences, 76 in total, were downloaded from NCBI GenBank nucleotide database (https://www.ncbi.nlm.nih.gov/nuccore) with the text search (ToxB) AND “Pyrenophora tritici-repentis”[porgn:__txid45151] (Additional file 3) and searched against the DW5 genome using BLATX v3.5 [39] (-maxIntron = 5000 -minIdentity = 70) and ≥ 50% query coverage (to detect any truncated genes).

Sequence flanking the identified *ToxB* loci, a total length of 10 kb, were then extracted using EMBOSS extractseq version 6.6.0.0 [40] and aligned with *ToxB* mRNA and CDS using Muscle [41] (-clwstrict). The multiple sequence alignment was then visualised in JalView version 2.10.5 [42], figures were created using the alignment overview.

To obtain a better view of sequence regions shared between the ten DW5 ToxB 10 kb regions, each sequence was aligned to each other at greater than 70% sequence identity, using BLAT version 3.5 [39] fastMap option, all coordinates were then used to create a bed file for visualisation using GenomeTools (gt) sketch version 1.5.10 [30].

The 2 kb sequence region upstream of *ToxB* was submitted to MEME Suite 5.1.1 [43] for motif discovery with classic discovery mode, site distribution zero or one occurrence and motif width between 6 and 50 inclusive. The most significant motif was submitted to TOMTOM [44] to identify similar motifs in the published nonredundant database JASPAR CORE 2018 [45] for eukaryotes.

### Whole genome alignment

DW5 PacBio assembled contigs were aligned to the optically mapped M4 chromosome scaffold [12] reference using NUCmer v3.1 (--maxmatch --coords) [46]. The sequence dot plot figure (Fig. 1) was generated using MUMmerplot v3.1 [46] with option for color plot line with percentage similarity gradient. EMBOSS revseq version 6.6.0.0 [40] was used for the reverse complementation of sequence.

The sequence dot plot of smaller regions (Fig. 3) were generated using Dotter version 4.44.1 [47].

The alignment and visualisation (Fig. 5) of the multiple *ToxB* loci regions for Contigs 9, 15, 16 and 18 was conducted using Easyfig (−blastn) linux version 2.2.2 [48].

## Supplementary information

**Additional file 1. **Nucleotide multiple sequence alignment of the ten *ToxB* loci regions.

**Additional file 2.** Predicted DNA binding site motif.

**Additional file 3.***Pyrenophora tritici-repentis* sequence accessions downloaded from NCBI GenBank nucleotide database (https://www.ncbi.nlm.nih.gov/nuccore) on the 27/02/2020.

## Data Availability

All data generated or analyzed during this study are included and can be accessed in this published article (and in Additional file 3). The annotated genome of DW5 has been deposited at DDBJ/ENA/GenBank repository under accession MUXC00000000. The DW5 version described in this paper is MUXC02000000. The annotated genome of M4 has been deposited at DDBJ/ENA/GenBank repository under accession NQIK00000000. The M4 version described in this paper is version NQIK02000000.
