# Correction to: PacBio genome sequencing reveals new insights into the genomic organisation of the multi-copy *ToxB* gene of the wheat fungal pathogen *Pyrenophora tritici-repentis*

**DOI:** 10.1186/s12864-021-07836-3

**Published:** 2021-08-09

**Authors:** Paula Moolhuijzen, Pao Theen See, Caroline S. Moffat

**Affiliations:** Centre for Crop Disease and Management, School of Molecular and Life Sciences, Curtin University, Perth, WA Australia

**Correction to: BMC Genomics 21, 645 (2020)**

**https://doi.org/10.1186/s12864-020-07029-4**

Following publication of the original article [1], it was reported that an update on the location of DW5 *ToxB* cluster relative to the M4 chromosome 10 fusion event was required.

Consequently, the Abstract, Table 2, Fig. 7 and several relevant sections of the article have been updated. The changes are given in this Correction article with the updated text highlighted in bold face.

**Table 2:** DW5 genome assembly relative to M4

M4 chromosome	M4 chromosome length (Mb)	DW5 contig	DW5 contig length (Mb)	DW5 contig 5’ and 3’ telomere motifs
1	9.91	1 12*	8.11 1.82	Yes
2	5.07	2	4.42	No
3	3.65	3	3.65	Yes
4	3.15	5	3.13	No
5	3.38	4*	3.36	Yes
6	3.05	6	2.73	No
7	2.76	13 11*	0.83 1.96	Yes
8	2.40	7	2.68	Yes
9	2.17	10	2.12	No
10	4.30	9*	2.18	No
**10**	**4.30**	8	2.18	Yes

*Reverse complemented sequence

**Fig. 7:**
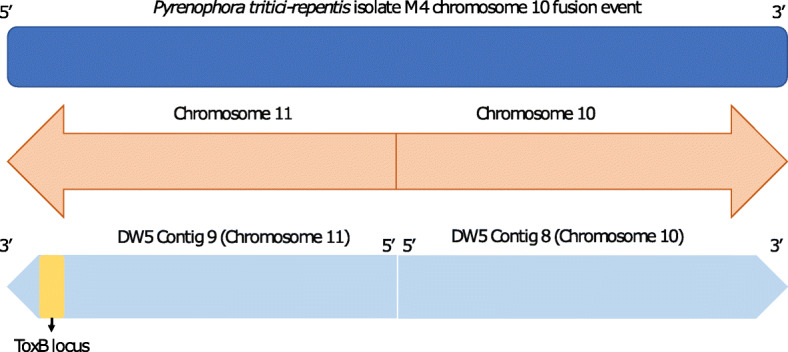
Overview of ToxB locus in DW5 relative to the M4 chromosome 10 fusion event. M4 chromosome 10 (top) is the result of a fusion between chromosomes 10 and 11 (shown in the middle). DW5 Contig 9 (chromosome 11) and Contig 8 (chromosome 10) (bottom) are shown relative to M4 chromosome 10. ToxB locus (yellow) which aligns to the 5’ distal region of M4 chromosome 10 is shown in the 3’ distal region of DW5 Contig 9 (chromosome 11)

**Abstract**

A total of ten identical *ToxB* gene copies were identified and based on flanking sequence identity, nine loci appeared associated with chromosome **11** and a single copy with chromosome 5. Chromosome **11** multiple *ToxB* gene loci were separated by large sequence regions between 31 - 66 kb within larger segmental duplications in an alternating pattern related to loci strand, and flanked by transposable elements.

**Whole genome comparative analysis between Ptr races 1 and 5**

A chromosome fusion between chromosome 10 and 11 (referred to as chromosome 10) in Australian isolate M4 resolved by optical mapping [2] was not observed for DW5, where DW5 contig 8 possessed both 5’ and 3’ telomere motifs (Table 2), which would represent **a** chromosome (telomere to telomere).

**Ptr ToxB multiloci analysis**

In this study, the *ToxB* loci were located on chromosome 5 and 11, which had assembly sizes of 3.36 and 2.18 Mb respectively, which are close to the previously estimated chromosome sizes by Martinez et. al, (2004). Of the ten *ToxB* loci, nine appeared to be associated with the smaller chromosome **11** located in the **3’** distal region. A Ptr chromosome noted for a chromosome fusion event for a race 1 isolate M4 [2]. The telomere to telomere support for eleven DW5 chromosomes is similar to the findings for another American race 1 isolate Ptr Pt-1C-BFP [3], unlike the 10 chromosome genome of Australian isolate M4 (1) (Fig. 7).

**Ptr ToxB patterns of duplication**

In addition to the positioning of the *ToxB* duplication within the distal region of chromosome **11**, *ToxB* loci were located equidistant downstream from dimer Tnp-haT transposases, a familiar gene found coupled to Ptr *ToxA* and within the horizontally transferred region, also found in *Parastagonospora nodorum* and *Bipolaris sorokiniana* [4, 5].
