# Supplementary figures and images for: PacBio genome sequencing reveals new insights into the genomic organisation of the multi-copy ToxB gene of the wheat fungal pathogen Pyrenophora tritici-repentis
Source: BMC Genomics. 2020 Sep 21;21:645. doi: 10.1186/s12864-020-07029-4 (PMC7507622; doi:10.1186/s12864-020-07029-4)

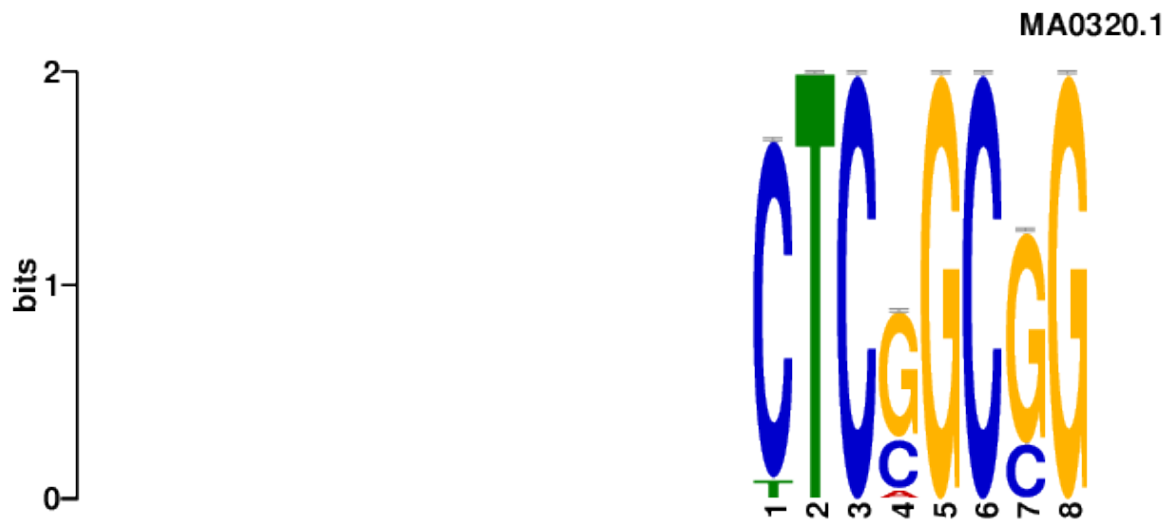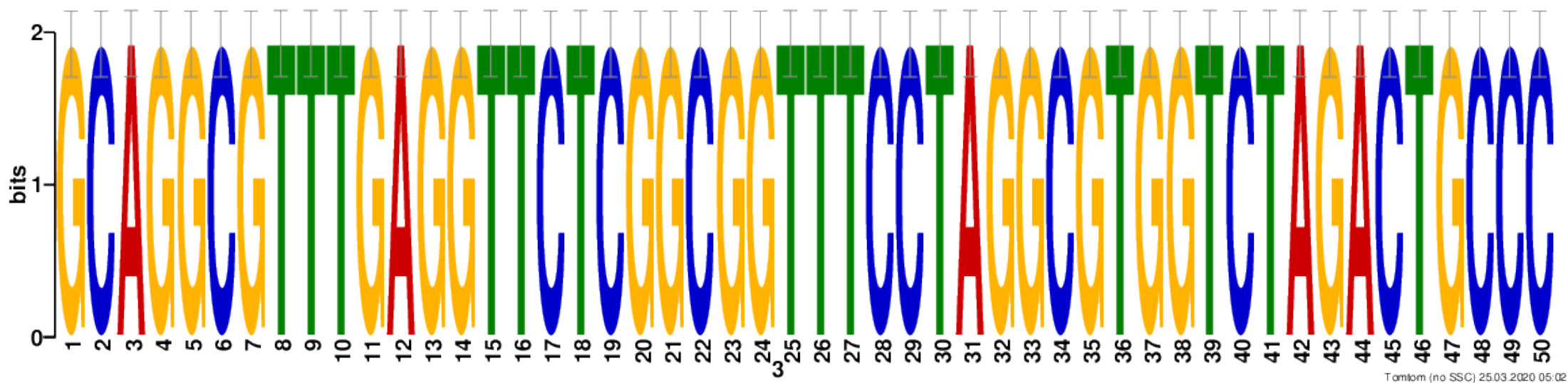

Supplement: Supplementary file 2 — Additional file 2. Predicted DNA binding site motif. [file 12864_2020_7029_MOESM2_ESM.pdf]
